# Fitness assessment to promote exercise for diabetes and hypertension

**DOI:** 10.6026/973206300220142

**Published:** 2026-01-31

**Authors:** Hulke S.M, Thakare A.E, Joshi A, Bharshankar R.N, Santosh Wakode

**Affiliations:** 1 Department of Physiology, All India Institute of Medical Sciences, Bhopal, India; 2 Department of Community and Family Medicine, All India Institute of Medical Sciences, Bhopal, India

**Keywords:** Diabetes mellitus, hypertension, fitness assessment, exercise prescription

## Abstract

Exercise adherence is a major problem in diabetic and hypertensive patients and fitness assessment can act as a tool to promote exercise adherence. With this objective, this study was planned for diabetic and hypertensive patients, involving two groups. One group was made aware of their fitness profile, while the other was not. Exercise adherence was tested by a Likert scale-based exercise adherence questionnaire score between the 2 groups, the Percentage of sessions completed as per the recommended duration irrespective of intensity/ type of exercise, the Percentage of sessions completed as per the prescribed intensity and the fitness profile comparison of the two groups at the end of 12 weeks. No significant differences were observed in the various parameters used to assess exercise adherence. Awareness of fitness assessment may not be considered a tool to promote exercise adherence; however further studies are recommended, whereby comparison with other methods of exercise adherence is done.

## Background:

About 537 million adults are estimated to be suffering from Diabetes mellitus in the age group of 20 and 79. This figure is continuously increasing and it is estimated that 783 million will have diabetes by the year 2045. In India, it is estimated that Incidence of diabetes will rise to 10.9% by the year 2045 [1]. Hypertension is not behind; the Fifth National Family Health Survey and the Indian Council of Medical Research have reported that it is increasing in India and more rapidly in rural and young populations [2]. Hypertension and diabetes are independent risk factors for each other. Both are considered to be deadly duos [3]. Exercise prescription is considered to be useful not only in diabetes mellitus and hypertension but also in various lifestyle disorders. During exercise prescription, care has to be given to prescribe intensity, time and type of activity [4]. An ideal exercise program should be specific, measurable, attainable, realistic and time-oriented. Such exercise prescription was found to be helpful in the diabetic population [5]. Various measures are mentioned in the literature to measure exercise adherence like diaries, logbooks, practice records, journals, surveys, questionnaires and scales. Diaries and logbooks were the most frequently used adherence measurement methods to monitor exercise adherence [6]. All the exercises ultimately have a positive effect on fitness parameters and these fitness parameter assessment is possible with various simple methods as well as application [7]. A fitness profile may be useful for a treating physician to monitor patients' fitness. Further, it would be helpful to counsel the patient for exercise. Therefore, it is of interest to describe a fitness assessment to promote exercise for diabetes and hypertension.

## Subjects and Methods:

This prospective study was done in the Physiology Department in collaboration with Medicine department, AIIMS, Bhopal. The study was conducted after departmental RRB approval and ethical clearance. The study was conducted on 20 patients diagnosed with diabetes mellitus with or without hypertension as per standard guidelines. Exclusion criteria were Uncontrolled Diabetes mellitus/ Hypertension [8], History of alcohol/drug dependence, Any medical condition impairing physical ability to exercise as per protocol. The diagnosed cases were started with treatment as advised by the Physician. They were enrolled for the study only after written informed consent. They were given a log book of the exercise program for 3 months and instructed on how to use the log book. Patients were randomly divided using computer-generated randomization into 2 groups: Group 1 (n=10) had undergone an exercise program with a fitness assessment. This group was made aware of the target and given the target to increase their fitness level and Group 2 (n=10) had undergone an exercise program without awareness of fitness profile.

## Exercise program:

The program was based on ACSM guidelines for Exercise in Diabetic and hypertensive populations. Patients were mainly prescribed exercise like walking/ brisk walking/ running five times a week. They were told the importance of exercise intensity and stepwise progression was advised. Patients were given the log book and were instructed to tick only if they had done exercise as recommended [9].

How would exercise adherence be monitored?

[1] Likert scale-based exercise adherence questionnaire score between 2 groups- based on score comparison. These questionnaires were prevalidated before being used in the patients.

[2] Percentage of sessions completed as per recommended duration irrespective of intensity/ type of exercise. Logbook data was used for this.

[3] Percentage of sessions completed as per prescribed intensity. Logbook data was used for this.

[4] Comparing fitness profile parameters of the two groups at the end of 12 weeks.

Fitness assessment parameters were as follows Table 1. For serial no. 1 standard guidelines would be followed. For serial 3 to 7, standard guidelines as mentioned in fit India guidelines (6) for the age group 18 to 65 years would be used. Handgrip and leg muscle strength was used by standard procedure [10]. Statistical analysis was done using statistical software. Wilcoxon signed rank test and unpaired t test were used to compare mean values of various parameters.

## Results and Discussion:

**Figure 1:**
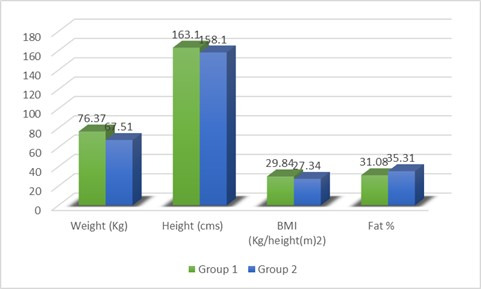
Physical characteristic and body composition parameters of two groups, Group 1 (n=10) and Group 2 (n=10)

**Figure 2:**
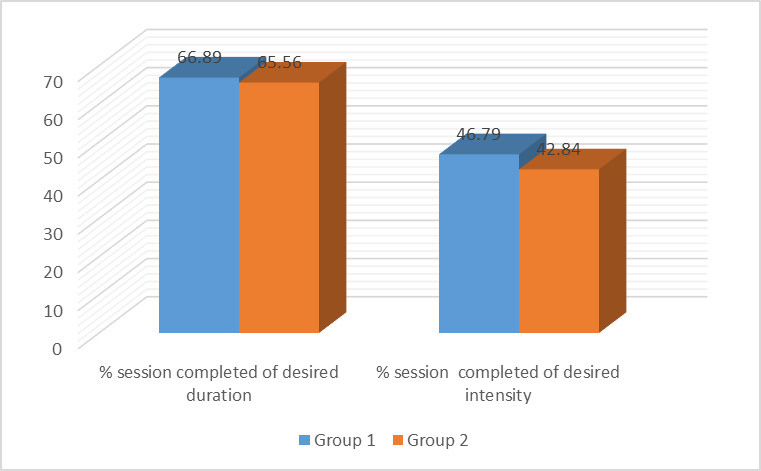
Difference in the percentage of sessions completed per duration and intensity.

The study was conducted in two groups, group 1; Age = 38.6 ± 7.02 yrs (Mean ± SD) (n=10, Male =7, Female= 3), group 2; Age = 39.9 ± 8.12 yrs (Mean ± SD) (n=10, Male =6, Female= 4). Physical characteristics and body composition of the subjects in 2 groups are shown in Figure 1, no significant difference was seen. Likert scale of Self-reported exercise adherence questionnaire of 2 groups is displayed, which also displayed no significant difference Table 2. A non-significant difference was also observed in the percentage of sessions completed as per the recommended duration and intensity between 2 groups Figure 2. Table 3 depicts various fitness parameters between 2 groups at the end of three months. No significant differences were observed between 2 groups. We have not come across any such studies to test exercise adherence. Various measures are mentioned in the literature to measure exercise adherence, including a diary, logbook, record of practice, journal, survey, questionnaire and scales. Diaries and logbooks were stroke patients' most frequently used adherence measurement methods [6]. There are various channels of communication for physical activity. One of the channels of communication is face-to-face intervention through physician contact. The tool used was individualized feedback based on the fitness profile of the patients. Besides face to face intervention, intervention through self- monitoring feedback devices may be helpful. Smartphone-based apps are helpful for this [11, 12]. There are various risk factors for non-adherence to exercise [13]. To overcome these problems, various strategies are targeted at building self-efficacy, brief counselling, motivational interviewing, interaction with exercise leaders (who do regular exercise) and cognitive behavioural approaches such as behavioural contracting, goal setting, self-monitoring and reinforcement [14]. Some of the effective change strategies are self-monitoring, planning and goal setting [15]. Exercise adherence through the technique described in present study can help for these skills.

Self-monitoring can be done by a fitness profile of oneself. Goal setting is one of the critical ways to promote exercise adherence. SMART principles for goal setting are specific, measurable, action-oriented, realistic, timely and self-determined [5]. In the Transtheoretical model of behavioral change, different people are in different stages of change and people pass through five stages: pre contemplation, contemplation, preparation, action and maintenance [16]. When the person is going through various stages of change as per the transtheoretical model, goal setting and self-monitoring to know whether there is any progress, play an important role [17]. This is possible with such a form of exercise adherence technique, especially in the contemplation and preparation stage. Levels of fitness profile knowledge may act as a tool for behavioural change in patients. Knowledge of fitness levels may be an intrinsic motivating factor for patients to engage in regular physical activity. Increased level of fitness parameters during exercise programs may act as an extrinsic reward, which will give the patient a sense of internal pleasure, thus promoting exercise adherence. Fitness profiles give knowledge about flexibility, muscular strength, core strength, muscular endurance and cardiovascular endurance. Assessing fitness profile is considered to be one of the prerequisites for pre-exercise testing and exercise prescription [10]. In a busy clinical setting in India, such an assessment may not be possible. However, these assessments are possible with minimal time with minimal infrastructure and in addition; the assessment can be done by the patient themselves. This assessment can act as a tool to assess the activity status of the patients and could help to give targets to the patients. However in the present case, no significant difference was seen between two groups. This may be mainly due less sample size and duration of the project. Still, fitness assessment may act as a tool to help treat physicians to be aware of the fitness profile of the patients and thereby prescribe individualized exercise plans. Patients can be explained how to assess their fitness level during follow-up. Thus, to conclude, awareness of fitness assessment may not be considered a tool to promote exercise adherence. However, further studies are recommended for comparison with other methods of fitness assessment.

## Figures and Tables

**Table 1:** Methods for fitness parameters assessment

**Sr. no.**	**Parameters**	**Method**	**Instruments**
1.	Body composition	Body mass index by measurement of height and weight, Fat percentage.	Weighing machine, height meter (stadiometer), Body composition analyser
2.	Flexibility	Sit and reach test	Sit and reach box
3.	Muscular strength Naukasan (boat pose)	Boat pose	Cushioned mat, stopwatch
4.	Abdominal/ core strength	Partial curl up	Cushioned mat, stopwatch
5.	Muscular endurance	Push up for males Modified push up for females	Cushioned mat
6.	Cardiovascular endurance	2 km run/walk test	lime powder, marker cone, stopwatch
7.	Static balance	Tree pose/ Vrikshasan Flamingo balance test	stopwatch
8.	Handgrip strength	Handgrip strength test	Handgrip dynamometer
9.	Leg muscle strength	Leg muscle strength test	Leg muscle dynamometer

**Table 2:** Likert scale of Self-reported exercise adherence questionnaire of 2 groups

**Sr No.**	**Questions**	**Group 1 (n=10)**	**Group 2 (n=10)**	**P value**
1.	I do my exercise for proper duration as recommended.	2.56±1.18	3.44±0.9	0.077
2.	I do my exercise with proper intensity as recommended.	2.8 ±0.89	3.54±0.97	0.09
3.	I do my exercise with frequency as recommended.	3.1±1.1	3.63±0.83	0.2
4.	I had increased duration and intensity of exercise over the entire period as recommended.	2.07±0.87	2.24±0.66	0.62
5.	I didn't do exercise as recommended but do exercise as it suites to me.	3.64±1.13	3.35±0.91	0.7
6.	I didn't do any form of exercise.	1.99±0.94	2.17±0.9	0.66
7.	I do exercise for frequency, duration and intensity less than that of recommended.	3.53±0.87	2.8±0.89	0.08
8.	I do exercise for frequency, duration and intensity more than that of recommended.	3.5±0.6	3.18±1.01	0.4

**Table 3:** Difference in Fitness parameters at the end of three months in two groups, Group 1 (n=10) and Group 2 (n=10)

**Fitness parameters**	**Group 1**	**Group 2**	**p-value**
Flexibility (cm)	20.7±4.65	21.8±6.76	0.67
Muscular Strength (sec)	30.6±7.46	30.2±16.71	0.94
Abdominal Strength (No.)	15.9±5.02	16.2±8.6	0.85
Muscular Endurance (No.)	11.1±5.86	11.1±7.1	1
Cardio-vascular Endurance (min)	21.55±3.38	21.5±4.43	0.98
Balance (Fall No.)	1.6±1.81	1.9±1.16	0.69
Balance (Sec)	45.7±13.42	44.9±13.52	0.9
Handgrip Strength (Kg)	47.54±12.07	47.32±6.84	0.66
Leg Muscle Strength (Kg)	60.45±14.13	61.9±11.33	0.82
